# A Novel Long Non‐Coding RNA lnc030 Maintains Breast Cancer Stem Cell Stemness by Stabilizing SQLE mRNA and Increasing Cholesterol Synthesis

**DOI:** 10.1002/advs.202204046

**Published:** 2022-08-25

**Authors:** Yilu Qin, Yixuan Hou, Shuiqing Liu, Pengpeng Zhu, Xueying Wan, Maojia Zhao, Meixi Peng, Huan Zeng, Qiao Li, Ting Jin, Xiaojiang Cui, Manran Liu*

*Adv. Sci*. **2021**, *8*, 2002232

DOI: 10.1002/advs.202002232

**Figure S6:**
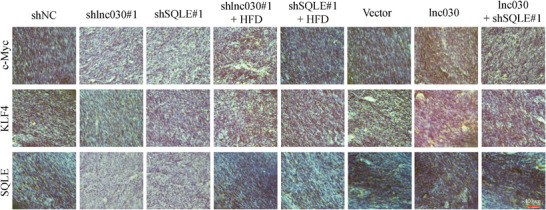
Lnc030 Promotes Breast Cancer Initiation and Progression (A). Representative immunohistochemical staining of SQLE, c‐Myc and KLF4 protein expression in tumor of xenografts (Scale bar, 100 µm).

In the original published Supporting Information of the article, some parts of the figure 6 were wrong. Please find below the correct Figure S6. The author apologize for any inconvenience caused.

